# Selective inhibition of MDR1 P-glycoprotein-mediated transport by the acridone carboxamide derivative GG918

**DOI:** 10.1038/sj.bjc.6690169

**Published:** 1999-03

**Authors:** A Wallstab, M Koester, M Böhme, D Keppler

**Affiliations:** Division of Tumor Biochemistry, Deutsches Krebsforschungszentrum, Im Neuenheimer Feld 280, D-69120, Heidelberg, Germany

**Keywords:** multidrug resistance (MDR), P-glycoprotein, ATP-dependent transport, transport inhibition, GG918 (formerly GF120918)

## Abstract

The acridone carboxamide derivative GG918 (N-{4-[2-(1,2,3,4-tetrahydro-6,7-dimethoxy-2-isoquinolinyl)-ethyl]-phenyl}-9,10-dihydro-5-methoxy-9-oxo-4-acridine carboxamide) is a potent inhibitor of MDR1 P-glycoprotein-mediated multidrug resistance. Direct measurements of ATP-dependent MDR1 P-glycoprotein-mediated transport in plasma membrane vesicles from human and rat hepatocyte canalicular membranes indicated 50% inhibition at GG918 concentrations between 8 nM and 80 nM using *N*-pentyl-[^3^H]quinidinium, [^14^C]doxorubicin and [^3^H]daunorubicin as substrates. The inhibition constant *K*_i_ for GG918 was 35 nM in rat hepatocyte canalicular membrane vesicles with [^3^H]daunorubicin as the substrate. Photoaffinity labelling of canalicular and recombinant rat Mdr1b P-glycoprotein by [^3^H]azidopine was suppressed by 10 μM and 40 μM GG918. The high selectivity of GG918-induced inhibition was demonstrated in canalicular membrane vesicles and by analysis of the hepatobiliary elimination in rats using [^3^H]daunorubicin, [^3^H]taurocholate and [^3^H]cysteinyl leukotrienes as substrates for three distinct ATP-dependent export pumps. Almost complete inhibition of [^3^H]daunorubicin transport was observed at GG918 concentrations that did not affect the other hepatocyte canalicular export pumps. The high potency and selectivity of GG918 for the inhibition of human MDR1 and rat Mdr1b P-glycoprotein may serve to interfere with this type of multidrug resistance and provides a tool for studies on the function of these ATP-dependent transport proteins. © 1999 Cancer Research Campaign


*British Journal of Cancer* (1999) **79**(7/8), 1053–1060
© 1999 Cancer Research Campaign
Article no. bjoc.1998.0169

### Selective inhibition of MDR1 P-glycoprotein-mediated transport by the acridone carboxamide derivative GG918

**A Wallstab, M Koester, M Böhme and D Keppler**

Division of Tumor Biochemistry, Deutsches Krebsforschungszentrum, Im Neuenheimer Feld 280, D-69120 Heidelberg, Germany

**Summary** The acridone carboxamide derivative GG918 (N-{4-[2-(1,2,3,4-tetrahydro-6,7-dimethoxy-2-isoquinolinyl)-ethyl]-phenyl}-9,10-dihydro-5-methoxy-9-oxo-4-acridine carboxamide) is a potent inhibitor of MDR1 P-glycoprotein-mediated multidrug resistance. Direct measurements of ATP-dependent MDR1 P-glycoprotein-mediated transport in plasma membrane vesicles from human and rat hepatocyte canalicular membranes indicated 50% inhibition at GG918 concentrations between 8 nM and 80 nM using *N*-pentyl-[$^3$H]quinidinium, [$^{14}$C]doxorubicin and [$^3$H]daunorubicin as substrates. The inhibition constant $K_i$ for GG918 was 35 nM in rat hepatocyte canalicular membrane vesicles with [$^3$H]daunorubicin as the substrate. Photoaffinity labelling of canalicular and recombinant rat Mdr1b P-glycoprotein by [$^3$H]azidopine was suppressed by 10 µM and 40 µM GG918. The high selectivity of GG918-induced inhibition was demonstrated in canalicular membrane vesicles and by analysis of the hepatobiliary elimination in rats using [$^3$H]daunorubicin, [$^3$H]taurocholate and [$^3$H]cysteinyl leukotrienes as substrates for three distinct ATP-dependent export pumps. Almost complete inhibition of [$^3$H]daunorubicin transport was observed at GG918 concentrations that did not affect the other hepatocyte canalicular export pumps. The high potency and selectivity of GG918 for the inhibition of human MDR1 and rat Mdr1b P-glycoprotein may serve to interfere with this type of multidrug resistance and provides a tool for studies on the function of these ATP-dependent transport proteins.

**Keywords:** multidrug resistance (MDR); P-glycoprotein; ATP-dependent transport; transport inhibition; GG918 (formerly GF120918)

The phenomenon of multidrug resistance may be caused by increased drug export mediated by adenosine 5′-triphosphate (ATP)-dependent export pumps of the plasma membrane (Gottesman and Pastan, 1993; Loe et al, 1996). The overexpression of the plasma membrane protein MDR1 P-glycoprotein (MDR1 P-gp) is one important mechanism leading to drug resistance (Juliano and Ling, 1976; Gottesman and Pastan, 1993). The P-gps are products of the *MDR* (multidrug resistance) gene family, but only the MDR1 protein in man (Roninson et al, 1986) and the Mdr1b and Mdr1a proteins in rodents (Gros et al, 1988; Devault and Gros, 1990) mediate ATP-dependent transport of cytotoxic P-gp substrates (Gottesman and Pastan, 1993). These substrates include several anticancer drugs that are hydrophobic and mostly cationic compounds (Pearce et al, 1989). Inhibition of the ATP-dependent export of the amphiphilic cationic anticancer drugs may serve to circumvent this type of multidrug resistance. Various drugs, including verapamil and other calcium channel blockers (Tsuruo et al, 1981), cyclosporins (Twentyman, 1992), as well as steroids (Yang et al, 1990) were found to enhance the intracellular accumulation and cytotoxic action of MDR1 P-gp-transported drugs (Ford and Hait, 1990). Most of these inhibitors bind to MDR1 P-gp and are themselves substrates of this transporter. Some of the MDR1 P-gp inhibitors used in clinical trials showed side-effects that restrict their therapeutic usefulness, e.g. because of cardiac toxicity (Tsuruo et al, 1981; Ford and Hait, 1990; Pennock et al, 1991) or immunosuppression (Twentyman, 1992). Multidrug resistance reversal agents may also affect ATP-dependent transporters in the liver. Cyclosporin A and its non-immunosuppressive derivative PSC 833 potently inhibit the bile salt export pump in the canalicular plasma membrane of hepatocytes (Böhme et al, 1993, 1994*a*, 1994*b*; Kadmon et al, 1993). The discovery of new and efficient MDR1 P-gp inhibitors with fewer side-effects is an important objective for future cancer chemotherapy. Effective chemosensitizers should be drugs that interact with MDR1 P-gp with high affinity and selectivity, thereby precluding its ability to extrude the cytotoxic drugs. A multidrug resistance reversal potency at nanomolar concentrations has been described for the acridone carboxamide derivative GG918 (Hyafil et al, 1993) and, more recently, for the cyclopropyldibenzosuberane modulator LY335979 (Dantzig et al, 1996). Such compounds may allow for an increased selectivity for inhibition of MDR1 P-gp-mediated membrane transport.

For studies on the selectivity of MDR1 P-gp inhibitors, the measurement of ATP-dependent transport by hepatocyte canalicular plasma membrane vesicles provides most useful approach (Böhme et al, 1993, 1994*a*). P-gps have been localized to the canalicular membrane of hepatocytes (Thiebaut et al, 1987; Kamimoto et al, 1989). Additional ATP-dependent export pumps that have been identified in this hepatocyte membrane domain include the ATP-driven bile salt export pump (BSEP), which secretes bile salts such as taurocholate (TC) into bile (Gerloff et al, 1998), and the ATP-dependent conjugate export pump encoded by the *MRP2* (*cMOAT*) gene which transports glutathione, glucuronate and sulfate conjugates of various exogenous and

*Received 1 June 1998*
*Revised 25 August 1998*
*Accepted 26 August 1998*

*Correspondence to:* D Keppler

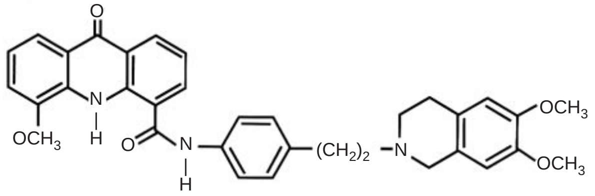

**Figure 1** Structure of the acridone carboxamide derivative GG918

endogenous lipophilic substances (reviewed by Keppler and König, 1997).

In the present study we determined the selectivity of the MDR1 P-gp inhibitor GG918 with respect to these different ATP-dependent transport systems in the hepatocyte canalicular membrane. The potency of GG918 was investigated in plasma membrane vesicles from human and rat. The inhibition of photoaffinity labelling by [$^{3}$H]azidopine in the presence of GG918 was demonstrated with recombinant rat Mdr1b P-gp. Moreover, GG918 was a very selective inhibitor for the in vivo hepatobiliary excretion of the MDR1/Mdr1b P-gp substrate [$^{3}$H]daunorubicin, whereas the excretion of [$^{3}$H]taurocholate and *N*-[$^{3}$H]acetyl-leukotriene $E_4$, which are substrates for the BSEP and MRP2, respectively, was not significantly affected.

### MATERIALS AND METHODS

### Materials

[$^{3}$H]Daunorubicin (57 GBq mmol$^{-1}$), [$^{3}$H]taurocholate (74 GBq mmol$^{-1}$), and [14,15,19,20-$^{3}H_4$]leukotriene (LT)$C_4$ (6.4 TBq mmol$^{-1}$) were purchased from Du Pont NEN, Bad Homburg, Germany. *N*-[$^{3}$H]Acetyl-$LTE_4$ (1.85 GBq mmol$^{-1}$) was synthesized in our laboratory using a chemical *N*-acetylation procedure (Guhlmann et al, 1995). *N*-(n-Pentyl)-[$^{3}$H]quinidinium was synthesized in our laboratory as described (Müller et al, 1994*a*). [$^{14}$C]Doxorubicin (211 GBq mmol$^{-1}$), [$^{3}$H]azidopine (1.96 TBq mmol$^{-1}$), and unlabelled $LTC_4$ were from Amersham Buchler, Braunschweig, Germany. GG918, formerly also designated GF120918, is an acridone carboxamide derivative (Hyafil et al, 1993) (Figure 1), was kindly provided by Dr F Hyafil from the GLAXO laboratories, Les Ulis, France. Ketamine was from Parke-Davis, Berlin, Germany. Xylazine was purchased from Bayer, Leverkusen, Germany. ATP, 5′-AMP, creatine phosphate potassium salt and unlabelled taurocholate were purchased from Sigma, Deisenhofen, Germany. Creatine kinase and reduced glutathione were obtained from Boehringer Mannheim, Mannheim, Germany. Percoll and NICK spin columns filled with Sephadex G-50 fine were from Pharmacia-LKB, freiburg, Germany. Glass microfibre filters (type GF/F, pore size ≥ 0.7 µm) were purchased from Whatman International Ltd., Maidstone, UK. Nitrocellulose filters (0.2 µm pore size) were from Schleicher & Schuell, Dassel, Germany. Scintillation fluids (Filter Count and Ultima Gold) were from Canberra Packard, Warrenville, IL, USA. Membranes of the recombinant *mdr1b*-BV-infected Sf9-cells were produced in our laboratory and kindly provided by Dr K Mittenbühler (Müller et al, 1994*b*).

### Animals

Male Wistar rats (200–250 g) were from the Charles River Wiga, Sulzfeld, Germany. Animals were maintained on an Altromin Nr. 1320 diet with free access to food and water.

### Human liver

Liver samples were obtained perioperatively from excised hepatic tissue from a patient suffering from primary hepatocellular carcinoma. Pathological tissue was removed, as estimated by macroscopic inspection, and only normal tumour-free liver tissue was further processed.

### Preparation and characterization of canalicular membranes

Membrane fractions enriched in the hepatocyte canalicular membrane domain were prepared and characterized from rat and human liver as described (Meier and Boyer, 1990; Kadmon et al, 1993; Böhme et al, 1994*b*).

### Measurement of ATP-dependent daunorubicin and doxorubicin transport into canalicular membrane vesicles

The [$^{14}$C]doxorubicin and [$^{3}$H]daunorubicin transport was measured by centrifugation of the vesicles through a gel matrix (Büchler et al, 1994). NICK spin columns (0.2 g Sephadex G-50/3.3 ml), rinsed with 250 mM sucrose, 10 mM Tris-HCl, pH 7.4, were centrifuged at 400 ***g*** for 4 min at 4°C. Membrane vesicles (30 µg protein) were incubated in the presence of 4 mM ATP, 10 mM magnesium chloride, 10 mM creatine phosphate, 100 µg ml$^{-1}$ creatine kinase, and the labelled substrate in 250 mM sucrose and 10 mM Tris-HCl (pH 7.4) at 37°C. The final volume was 110 µl. The standard substrate concentration was 20 µM. At the indicated time points, 20 µl aliquots were taken and diluted in 80 µl ice-cold Tris buffer (250 mM sucrose, 10 mM Tris-HCl, pH 7.4) and immediately loaded on Sephadex G-50 columns. The columns were eluted with 100 µl ice-cold Tris buffer and centrifuged at 400 ***g*** for 4 min at 4°C. The membrane vesicle-containing effluent was collected in scintillation vials and counted for radioactivity. In control experiments ATP was replaced by 5′-AMP. Transport rates were calculated by subtracting the values in the presence of 5′-AMP from those in the presence of ATP.

### Measurement of ATP-dependent *N*-pentyl-quinidinium transport into canalicular membrane vesicles

The transport of *N*-(n-pentyl)-[$^{3}$H]quinidinium was measured by the rapid filtration technique using glass microfibre filters (pore size ≥ 0.7 µm) (Müller et al, 1994*a*). The filters were presoaked in Tris-buffered saline (pH 7.4). This filtration was performed at a pressure of 850 mbar. Membrane vesicles were incubated in the standard incubation mixture as described above for [$^{3}$H]daunorubicin. The concentration of *N*-pentyl-[$^{3}$H]quinidinium was 1 µM. Samples of 20 µl were taken at the indicated time-points and diluted in 950 µl of ice-cold Tris-buffered saline (pH 7.4). This solution was applied to the microfibre filters and immediately rinsed with 5 ml of ice-cold Tris-buffered saline (pH 7.4) containing 0.05% (v/v) Tween 20 and 5 ml of Tris-buffered saline (pH 7.4). Vesicle-associated radioactivity retained on the filters was assessed by liquid scintillation counting.

### Measurement of ATP-dependent taurocholate transport into canalicular membrane vesicles

Transport of [$^{3}$H]taurocholate was measured by a rapid filtration technique using nitrocellulose filters (0.2 µm pore size) presoaked

*© Cancer Research Campaign 1999*

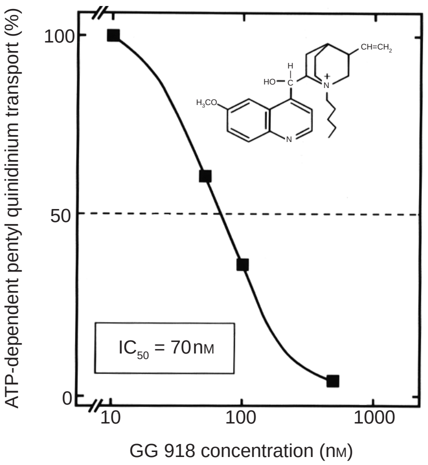

**Figure 2** Inhibition by GG918 of the ATP-dependent *N*-pentyl-[$^3$H]quinidinium transport by human canalicular membrane vesicles. The vesicles were incubated as described under Materials and methods at different inhibitor concentrations for 3 min at 37°C. In the blank assays ATP was replaced by 5′-AMP. The concentration of *N*-pentyl-[$^3$H]quinidinium was 1 μM; the structure of this substrate (Müller et al, 1994*a*) is shown in the upper right corner. The data for each inhibitor concentration represent percentage inhibition of transport as compared to controls in the absence of inhibitor. Each point represents the mean of four measurements

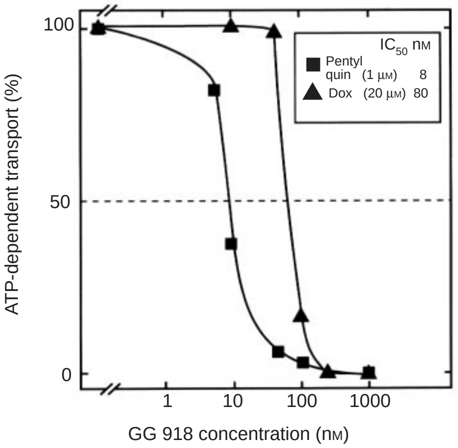

**Figure 3** Inhibition of the Mdr1 P-gp-mediated ATP-dependent transport by GG918 in rat hepatocyte canalicular membrane vesicles. The vesicles were incubated with 20 μM [$^{14}$C]doxorubicin and 1 μM *N*-pentyl-[$^3$H]quinidinium in the presence of the inhibitor, at the concentrations indicated, for 3 min at 37°C. The data for each inhibitor concentration represent percentage inhibition of transport as compared to controls in the absence of inhibitor. The values represent the mean of four determinations in 250 mM sucrose, 10 mM Tris-HCl (pH 7.4) (Böhme et al, 1993). Membrane vesicles (30 μg of protein) were incubated as described above for the MDR1 P-gp substrates. Aliquots (20 μl) were taken at the indicated time-points, diluted in 1 ml of ice-cold incubation buffer and applied to the nitrocellulose filters. The filters were rinsed with 5 ml washing buffer containing 1 mM unlabelled taurocholate, 250 mM sucrose, 10 mM Tris-HCl (pH 7.4) and 5 ml buffer consisting of 250 mM sucrose, 10 mM Tris-HCl (pH 7.4). In control experiments ATP was replaced by 5′-AMP. Vesicle-associated radioactivity retained on the filters was assessed by liquid scintillation counting.

### Measurements of ATP-dependent LTC$_4$ transport into canalicular membrane vesicles

[$^3$H]LTC$_4$ transport was determined as described recently (Keppler et al, 1998). The rinsing buffer consisted of 250 mM sucrose and 10 mM Tris-HCl (pH 7.4). Reduced glutathione was added to the incubation at a final concentration of 5 mM to prevent binding of [$^3$H]LTC$_4$ to membrane-bound glutathione *S*-transferase and to inhibit degradation of [$^3$H]LTC$_4$ to [$^3$H]LTD$_4$ by the canalicular membrane γ-glutamyltransferase.

### Photoaffinity labelling with [$^3$H]azidopine

Membrane vesicle suspensions (75–100 μg protein) were diluted in 250 mM sucrose, 10 mM Tris-HCl (pH 7.4) and incubated with [$^3$H]azidopine (74 kBq, 1.2 μM) at 25°C for 25 min in the presence of 0.5% fetal calf serum. The membrane solution was irradiated at 254 nm for 2 min on ice. For the competition studies, vesicle suspensions were pre-incubated with 10 μM or 40 μM of GG918 for 25 min at 25°C. The labelled membranes were pelleted by centrifugation for 30 min at 100 000 ***g*** at 4°C. The pellet was resuspended in Laemmli buffer (Laemmli, 1970) and subjected to sodium dodecyl sulphate polyacrylamide gel electrophoresis (SDS-PAGE) (7.5% acrylamide gel). After electrophoresis the gels were cut into slices of 2 mm thickness and dissolved in 0.5 ml of the tissue solubilizer Biolute-S (Zinsser Analytic, Frankfurt, Germany). Radioactivity was measured in a liquid scintillation counter.

### In vivo elimination of daunorubicin, taurocholate and *N*-acetyl-leukotriene E$_4$ into bile

Rats were anaesthetized by intraperitoneal injection of ketamine (80 mg kg$^{-1}$) and xylazine (12 mg kg$^{-1}$). The cannulation of the common bile duct and of the superior vena cava through the jugular vein was performed while rats were under general anaesthesia. The bile flow was allowed to reach a steady-state for 30 min and the experiment was started by intravenous injection of 2.2 mg kg$^{-1}$ body weight (4.0 μmol kg$^{-1}$) of GG918. The stock solution of GG918 was 4 mg/m (dissolved in PEG 400/$H_2O$ 1:1; v/v) corresponding to a 7.1 mM solution. The inhibitor was diluted twofold in pyrogen-free distilled water and slowly injected over a 1-min period. [$^3$H]Daunorubicin (185 kBq kg$^{-1}$; 3.2 nmol kg$^{-1}$ body weight), [$^3$H]taurocholate (148 kBq kg$^{-1}$; 2 nmol kg$^{-1}$ body weight), or *N*-[$^3$H]acetyl-LTE$_4$ (185 kBq kg$^{-1}$; 100 nmol kg$^{-1}$ body weight) were injected as a bolus 10 min later. Bile was collected in 5-min fractions for 90 min, and the radioactivity in these samples was determined by diluting aliquots of bile in 100% methanol. Supernatants of the methanol extract were dissolved in 10 ml of scintillation fluid and counted for radioactivity. In the control group, GG918 was replaced by a corresponding volume of the vehicle (PEG 400/$H_2O$ 1:1; v/v; diluted as described above).

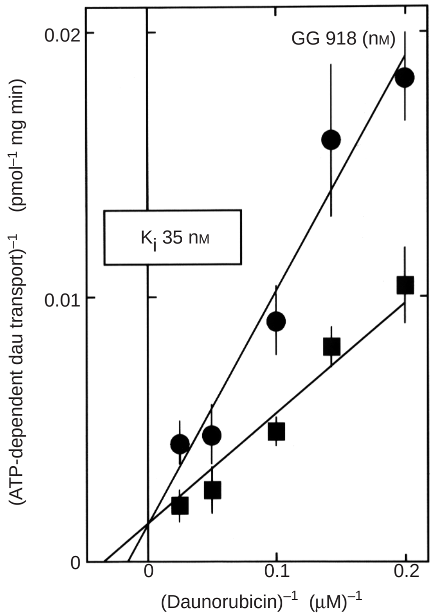

**Figure 4** Inhibition of ATP-dependent [³H]daunorubicin transport into rat hepatocyte canalicular membrane vesicles by GG918. Bile canalicular membranes (30 μg) were preincubated in the presence of 30 nM GG918 (●) or the corresponding volume of the solvent (■) for 1 min at 37°C with 0.5% fetal calf serum. Transport was started by the addition of 4 mM ATP (or 5′-AMP in the control experiment), 10 mM creatine phosphate, 10 mM magnesium chloride and varying [³H]daunorubicin concentrations. Double reciprocal plot according to Lineweaver and Burk. Mean values ± SD from three measurements

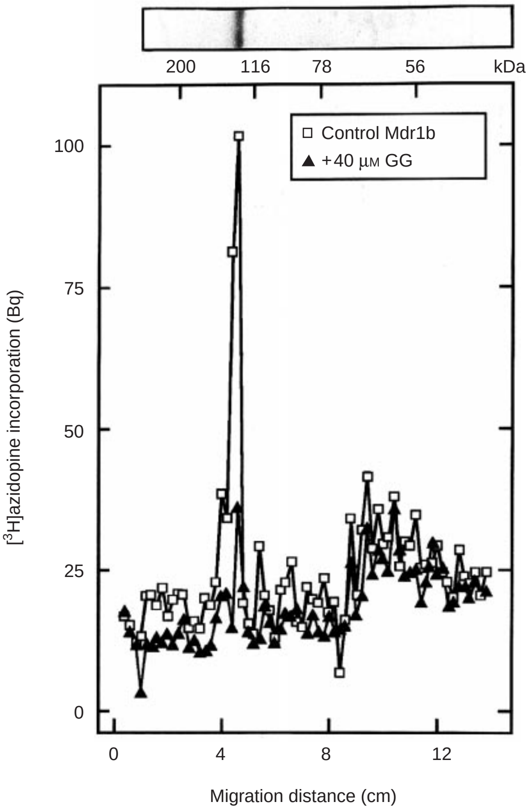

**Figure 5** [³H]Azidopine binding of the 140 kDa Mdr1b P-gp in membranes of recombinant BV-infected Sf9 insect cells identified by photoaffinity labelling and its suppression by the inhibitor GG918. The distribution of the [³H]azidopine-derived radioactivity was studied after SDS-PAGE of the photoaffinity labelled membranes. For the labelling the membranes (75 μg of protein) of Mdr1b-expressing cells (control Mdr1b; □) were incubated with 74 kBq [³H]azidopine as described in Materials and Methods. In addition, the [³H]azidopine labelling of Mdr1b containing membranes was performed in the presence of 40 μM GG918 (+ 40 μM GG; ▲). The top lane shows the immunodetection of the recombinant Mdr1b after SDS-PAGE using the C219 antibody

### RESULTS

### Kinetic characterization of the inhibition by GG918 of transport of MDR1 and Mdr1b P-gp substrates in human and rat hepatocyte canalicular membrane vesicles

Initial experiments compared the inhibitory effect of GG918 on ATP-dependent transport in canalicular membranes isolated from human and rat liver. *N*-(n-Pentyl)-[³H]quinidinium, a quinidinium compound with a permanently charged nitrogen atom, which served as a high affinity substrate for MDR1 P-gp-mediated transport (Müller et al, 1994*a*, 1994*b*). The $IC_{50}$ value for GG918 in human hepatocyte canalicular membranes was 70 nM at a *N*-(n-pentyl)-[³H]quinidinium concentration of 1 μM (Figure 2). The ATP-dependent *N*-(n-pentyl)-[³H]quinidinium transport was reduced to 36% by 100 nM GG918. In rat hepatocyte canalicular membranes the half-maximal inhibition of *N*-pentyl-[³H]quinidinium transport by GG918 was observed at a concentration of 8 nM (Figure 3) indicating a more potent inhibition of the rat liver transporter as compared to human MDR1 P-gp (Figure 2). Concentrations of 100 nM and 10 nM GG918 suppressed ATP-dependent transport of *N*-pentyl-[³H]quinidinium by 98% and 42%, respectively (Figure 2). [¹⁴C]Doxorubicin transport, measured at a substrate concentration of 20 μM, was half-maximally inhibited by 80 nM GG918 (Figure 3).

Transport of [³H]daunorubicin into inside-out oriented vesicles from rat hepatocyte canalicular membranes was inhibited by GG918 in a competitive manner (Figure 4). The kinetic constants for the ATP-dependent daunorubicin transport were determined by Lineweaver–Burk plots and yielded an apparent $K_m$ of 33 μM and a $V_{max}$ of 721 pmol mg⁻¹ min⁻¹. The $K_i$ value for GG918 was 35 nM for [³H]daunorubicin transport (Figure 4).

### Photoaffinity labelling with [³H]azidopine

Membranes of recombinant Mdr1b-expressing Sf9 insect cells were used to show the specific affinity of azidopine to P-gp in photoaffinity labelling experiments. Membrane vesicles of recombinant *mdr1b*-BV-infected Sf9 cells were exposed to [³H]azidopine and revealed one major band at 140 kDa after irradiation and consecutive separation by SDS-PAGE (Figure 5). As shown by C219 anti-P-gp antibody detection of this membrane preparation the labelled protein co-migrated with recombinant Mdr1b. Photoaffinity labelling in the presence of 40 μM GG918 caused an 80% reduction of [³H]azidopine incorporation into the Mdr1b protein. In these insect membranes we observed a very low unspecific binding of [³H]azidopine. In contrast, in rat canalicular membranes several [³H]azidopine-labelled proteins were detected (Figure 6). Only the incorporation of radioactivity in the 145 kDa protein, which corresponds to a C219 antibody-detectable P-gp, was decreased by preincubation with GG918 in a dose-dependent manner (Figure 6). The presence of 10 μM and 40 μM GG918 reduced the [³H]azidopine labelling of rat P-gp by 38% and 78%, respectively.

© Cancer Research Campaign 1999

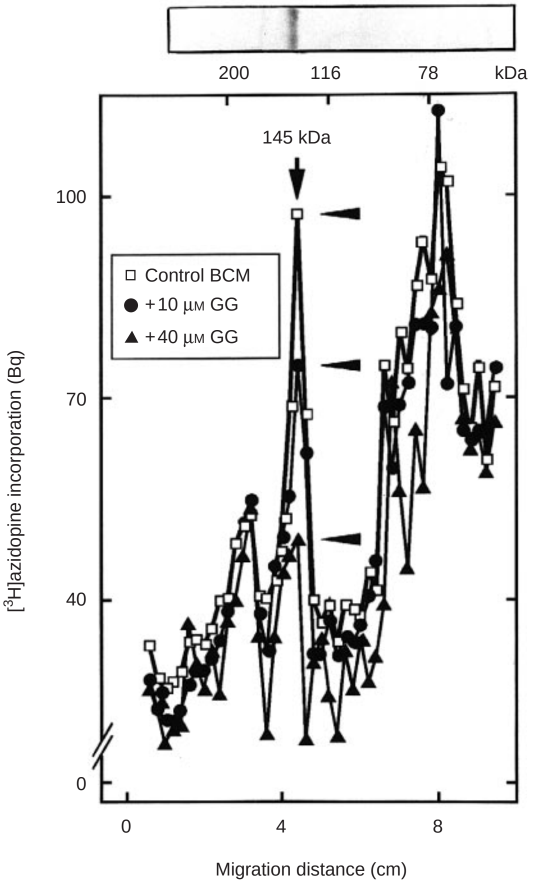

**Figure 6** [$^3$H]Azidopine labelling of Mdr1 P-gp in rat canalicular membranes and its suppression by GG918. Hepatocyte canalicular membranes (100 μg of protein) were incubated with 74 kBq [$^3$H]azidopine in the absence (control BCM, □) or presence of 10 μM (10 μM GG, ●) or 40 μM (40 μM GG, ▲) GG918 at 25°C for 25 min. The samples were photoaffinity labelled as described in Materials and methods and separated by SDS-PAGE using a 7.5% acrylamide gel. The peak at 145 kDa corresponds to the C219 antibody-detected Mdr P-gp. Arrows on the right of the 145 kDa peak indicate the decreased labelling of the 145 kDa protein by [$^3$H]azidopine in the presence of 10 μM GG918 and 40 μM GG918

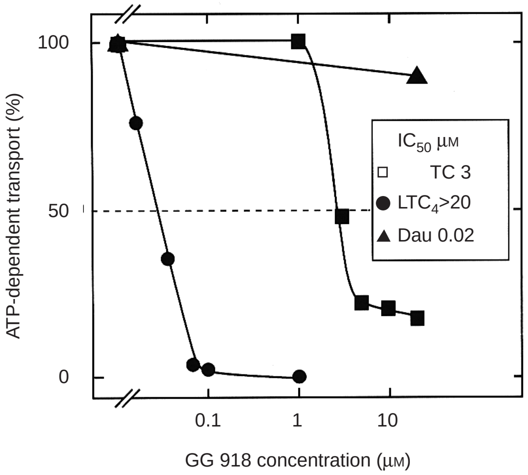

**Figure 7** Inhibition by GG918 of the ATP-dependent [$^3$H]daunorubicin (DAU), [$^3$H]LTC$_4$ and [$^3$H]taurocholate (TC) transport in rat hepatocyte canalicular membrane vesicles. The concentrations of TC (■), LTC$_4$ (▲) and DAU (●) were 5 μM, 50 nM and 20 μM, respectively. Uptake into plasma membrane vesicles (30 μg protein) was measured in the presence of 4 mM ATP. Vesicles were pre-incubated at 37°C for 1 min (30 s in case of TC as the transport substrate) in the presence of varying GG918 concentrations and 0.5% fetal calf serum before the addition of the labelled substrate. Each point represents the mean from three measurements

### In vitro inhibition of three different ATP-dependent export pumps in hepatocyte canalicular membranes by GG918

In inside-out oriented vesicles of rat canalicular membranes [$^3$H]LTC$_4$ transport was not significantly inhibited at GG918 concentrations up to 20 μM. The ATP-dependent BSEP, determined with [$^3$H]taurocholate as substrate, was inhibited by 50% at a GG918 concentration of about 3 μM. The high selectivity of the inhibitor for Mdr1b P-gp was indicated by a half-maximal inhibition at about 20 nM GG918 at a [$^3$H]daunorubicin concentration of 20 μM (Figure 7).

### Effect of GG918 on the hepatobiliary excretion of substrates for different ATP-dependent export pumps in vivo

The excretion of intravenously administered [$^3$H]daunorubicin, [$^3$H]taurocholate and *N*-[$^3$H]acetyl-LTE$_4$ was determined by analyses in bile. The bile flow was not affected by GG918 at a dose of 4 μmol kg$^{-1}$ body weight. The biliary recovery of [$^3$H]daunorubicin (administered at a dose of 3.2 nmol kg$^{-1}$ body weight) after 90 min was 9.7 ± 1.6% ($n$ = 4; mean ± SD) in control experiments without GG918 (Figure 8). GG918 pretreatment 10 min before tracer injection reduced this biliary excretion of [$^3$H]daunorubicin to 29% of the control. The cumulative biliary elimination of this tracer after GG918 pretreatment was 2.8 ± 0.3% of the injected dose. In a single experiment (data not shown) the dose of the inhibitor was raised from 4 to 14 μmol kg$^{-1}$ body weight. This resulted within the initial 30 min in a complete suppression of the [$^3$H]daunorubicin elimination into bile. After 90 min the cumulative secretion of [$^3$H]daunorubicin was reduced to 13% of control corresponding to a total recovery of 1.3% of the administered radioactivity after the high dose of GG918. In addition, [$^{14}$C]doxorubicin (175 pmol kg$^{-1}$ body weight; 37 kBq kg$^{-1}$) was injected to rats with or without GG918 (14 μmol kg$^{-1}$ body weight; data not shown). The biliary recovery of [$^{14}$C]doxorubicin was 11.4% after 60 min in untreated animals. GG918 reduced the recovery to 3.0 ± 0.5% ($n$ = 4; mean ± SD) of the injected dose corresponding to a suppression of the [$^{14}$C]doxorubicin elimination to 27%.

*N*-Acetyl-LTE$_4$ is the major metabolite of endogenous cysteinyl leukotrienes in rat bile (Denzlinger et al, 1985) and a substrate for the ATP-dependent conjugate export pump MRP2 in the hepatocyte canalicular membrane (Ishikawa et al, 1990). *N*-[$^3$H]acetyl-LTE$_4$ was therefore chosen in our experiments as the substrate for this export pump. No significant inhibition of *N*-[$^3$H]acetyl-LTE$_4$ secretion was observed after administration of GG918 (4 μmol kg$^{-1}$). The biliary recovery of intravenously administered *N*-[$^3$H]acetyl-LTE$_4$ (100 nmol kg$^{-1}$ body weight) was 93 ± 4% ($n$ = 4; mean ± SD) of the biliary secretion in animals without GG918. Moreover, the injection of GG918 (4 μmol kg$^{-1}$ body weight) did not affect the biliary excretion of [$^3$H]taurocholate (2 nmol kg$^{-1}$ body weight) as the major substrate of the BSEP (Figure 8).

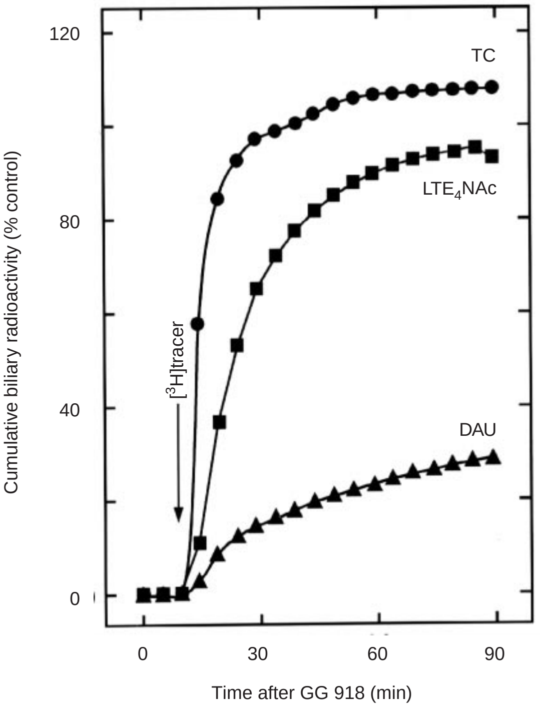

**Figure 8** Cumulative biliary recovery of the intravenously injected tracer doses of [$^3$H]taurocholate (TC; ●), *N*-[$^3$H]acetyl $LTE_4$ ($LTE_4$NAc; ■) and [$^3$H]daunorubicin (DAU; ▲) after 4 μmol GG918 per kg body weight over 90 min. The recovery is given as percent recovery compared to the corresponding control without GG918. The time of tracer administration is indicated by the arrow. Each point represents the mean of four experiments. The mean biliary recovery of [$^3$H]TC, *N*-[$^3$H]acetyl $LTE_4$, and [$^3$H]DAU in GG918-treated animals was 107, 93 and 30% of the untreated controls, respectively

### DISCUSSION

The ability to reverse one important type of multidrug resistance in cancer patients by inhibition of MDR1 P-gp-mediated drug transport by chemosensitizing agents is limited by the extent of toxic side-effects and by the selectivity of the MDR1 P-gp-inhibitor. Verapamil and cyclosporin A are well characterized examples of MDR1 P-gp-modulating compounds (Watanabe et al, 1995). However, verapamil has cardiac side-effects and lowers the blood pressure (Pennock et al, 1991), and the use of cyclosporin A is limited by its immunosuppressive action (Twentyman, 1992) and by its inhibition of ATP-dependent export pumps other than MDR1 P-gp (Böhme et al, 1993, 1994*a*, 1994*b*; Kadmon et al, 1993). In the present study we have determined the potency of the acridone carboxamide derivative GG918 for inhibition of three different ATP-dependent export pumps in the hepatocyte canalicular membrane:

1. The rat *mdr1b* gene-encoded multidrug resistance export pump
2. The BSEP, encoded by the so called sister of P-glycoprotein (spgp; Gerloff et al, 1998)
3. The *MRP2 (cMRP/cMOAT)* gene-encoded conjugate export pump (Keppler and König 1997).

Among these ATP-dependent transporters for daunorubicin, as substrate for Mdr1b P-gp (Kamimoto et al, 1989), taurocholate as substrate for the BSEP (Adachi et al, 1991; Müller et al, 1991; Nishida et al, 1991; Gerloff et al, 1998), and $LTC_4$ as well as *N*-acetyl-$LTE_4$, as substrates for Mrp2 (Ishikawa et al, 1990; Keppler and König, 1997), the Mdr1b P-gp export pump exhibited the highest susceptibility to inhibition by GG918 in vitro and in vivo (Figures 7 and 8). In comparison to the previously reported MDR1 P-gp inhibitors, except for the recently described modulator LY335979 (Dantzig et al, 1996), inhibition of the ATP-dependent transport of several structurally different MDR1 P-gp substrates was observed at a lower concentration of GG918, and half-maximal inhibition was detected between 8 nM and 80 nM (Figures 2, 3 and 7). This was shown for daunorubicin and doxorubicin as well as for the more hydrophilic *N*-pentyl-quinidinium. The different $IC_{50}$ values for the substrate *N*-pentyl-[$^3$H]quinidinium in human (70 nM) and rat liver canalicular membranes (8 nM) may be due to the species difference of the MDR1 and Mdr1b transporters with an amino acid sequence identity of 81% (Brown et al, 1993). Cyclosporin A is less specific as a MDR1 P-gp inhibitor and affects other ATP-dependent export pumps, particularly the ATP-dependent BSEP (Böhme et al, 1993). In the rat, the inhibition of the latter pump by cyclosporin A induces intrahepatic cholestasis (Böhme et al, 1994*a*, 1994*b*). PSC 833, a non-immunosuppressive cyclosporin A derivative (Boesch et al, 1991) also inhibits several other ATP-dependent export pumps (Böhme et al, 1993, 1994*a*) and is not as potent as GG918 with respect to inhibition of MDR1/Mdr1b P-gp (Figures 2–4). The inhibitory efficiency estimated by the $K_m/K_i$ ratio was 942 for GG918 when daunorubicin was used as the substrate. This value is 14-fold higher than the ratio for PSC 833 and 70-fold higher than this ratio for cyclosporin A (Böhme et al, 1993). GG918 does not seem to cause cytotoxicity in the concentrations required for modulation of multidrug resistance and unspecific toxic side-effects have not been observed at concentrations which were 100-fold above those necessary for specific MDR1 P-gp inhibition (Hyafil et al, 1993).

Many modulators of the MDR1 P-gp-mediated multidrug resistance are lipophilic and heterocyclic compounds with a basic nitrogen atom at physiological pH (Ford et al, 1989; Pearce et al, 1989; Hait and Aftab, 1992). The acridone carboxamide derivative GG918 exhibits these properties as well (Figure 1) and shows a high potency for inhibition of *N*-pentyl-quinidinium and daunorubicin transport (Figures 2–4). The different $IC_{50}$ values of GG918 for three different ATP-dependent export pumps of the canalicular membrane offers the possibility to inhibit the MDR1 P-gp export pump at a concentration of GG918 at which the conjugate and bile salt export pumps are not or only little affected. Our studies in vitro are in line with our in vivo studies in the rat where GG918 at the dose of 2 mg $kg^{-1}$ influenced only the Mdr1b P-gp-mediated excretion into bile (Figures 7 and 8). The similar bile flow rates of control and GG918-treated animals indicate that the processes involved in bile formation are not affected by this dose of the inhibitor. A higher dose of GG918 (8 mg $kg^{-1}$) caused a nearly complete suppression of the [$^3$H]daunorubicin secretion into bile during the initial 30 min after injection of GG918. This dose is in a range similar as the one which increased the survival time of tumour-bearing mice undergoing chemotherapy (Hyafil et al, 1993).

In photoaffinity labelling studies with [$^3$H]azidopine we demonstrated an inhibition of the labelling by GG918 that is consistent with the high affinity of GG918 to Mdr1b P-gp in hepatocyte canalicular membranes (Figure 6). GG918 also competed with [$^3$H]azidopine for binding to the rat Mdr1b P-gp overexpressed in Sf9 cells (Figure 5). The low level of non-specific labelling in membranes from Sf9 cells shows the suitability of such membranes for these experiments. On the other hand, hepatocyte canalicular membranes showed non-specific binding of

*© Cancer Research Campaign 1999*

[3H]azidopine to several proteins that was not competed for by GG918 (Figure 6). Our kinetic studies indicated a competitive inhibition of [3H]daunorubicin transport by GG918 (Figure 4). These results are compatible with the assumption that GG918 binds with high affinity to the substrate binding site of Mdr1b P-gp. The concentration of GG918 required for potent competition with [3H]azidopine photoaffinity labelling was at least 100-fold higher than the concentration necessary for potent inhibition of substrate transport by Mdr1b P-gp. While it is not possible to compare the kinetic processes during photoaffinity labelling with the kinetics of ATP-dependent membrane transport, it should be noted that suppression of photoaffinity labelling usually requires much higher concentrations than inhibition of transport. This may be exemplified by the suppression of photoaffinity labelling by [3H]$LTC_4$ with the potent transport inhibitor MK571 (Nicholson et al, 1992; Leier et al, 1994), or by the competitive photoaffinity labelling of intestinal bile salt binding sites using 7, 7-azo-taurocholate (Lin et al, 1990).

Highly potent inhibition of MDR1/Mdr1b P-gp may cause an impaired detoxification of substances in non-malignant P-gp-expressing tissues. As shown for the *mdr1a* gene knock-out mice, a total loss in Mdr1a function may lead to an entry of toxic substances into normal tissues such as the brain (Schinkel et al, 1994). Accordingly, potent inhibition of MDR1 P-gp in the blood–brain barrier may be followed by an action of toxic and non-toxic compounds on the brain that is prevented under normal conditions by the function of MDR1 P-gp. Such inhibition may be desirable, however, not only during chemotherapy of brain tumours, but also during antibacterial chemotherapy with substances that are substrates for MDR1 P-gp in the blood–brain barrier. The high potency and selectivity of a compound like GG918 for MDR1 P-gp not only offers a chance to reverse this type of multidrug resistance but also to study the physiological role of P-gp in several species.

### ACKNOWLEDGEMENTS

We are indebted to Dr F Hyafil, Les Ulis, France, for providing the inhibitor GG918. Drs JA Silverman and SS Thorgeirsson, Bethesda, MD, USA, kindly provided the cDNA encoding the rat Mdr1b P-glycoprotein. We thank Dr E Klar from the Surgery Department of Heidelberg University for his support in obtaining human liver samples, and Drs G Jedlitschky and I Leier from our laboratory in Heidelberg for their helpful advice during the course of these studies. This work was supported in part by grants from the Deutsche Forschungsgemeinschaft through SFB 352 and by the Forschungsschwerpunkt Transplantation Heidelberg.

### REFERENCES

Adachi Y, Kobayashi H, Kurumi Y, Shouji M, Kitano M and Yamamoto T (1991) ATP-dependent taurocholate transport by rat liver canalicular membrane vesicles. *Hepatology* **14**: 655–659

Böhme M, Büchler M, Müller M and Keppler D (1993) Differential inhibition by cyclosporins of primary-active ATP-dependent transporters in the hepatocyte canalicular membrane. *FEBS Lett* **333**: 193–196

Böhme M, Jedlitschky G, Leier I, Büchler M and Keppler D (1994a) ATP-dependent export pumps and their inhibition by cyclosporins. *Adv Enzyme Regul* **34**: 371–380

Böhme M, Müller M, Leier I, Jedlitschky G and Keppler D (1994b) Cholestasis caused by inhibition of the adenosine triphosphate-dependent bile salt transport in rat liver. *Gastroenterology* **107**: 255–265

Boesch D, Gaveriaux C, Jachez B, Pourtier-Manzanedo A, Bollinger P and Loor F (1991) In vivo circumvention of P-glycoprotein-mediated multidrug resistance of tumor cells with SDZ PSC 833. *Cancer Res* **51**: 4226–4233

Brown PC, Thorgeirsson SS and Silverman JA (1993) Cloning and regulation of the rat *mdr2* gene. *Nucleic Acids Res* **21**: 3885–3891

Büchler M, Böhme M, Ortlepp H and Keppler D (1994) Functional reconstitution of ATP-dependent transporters from the solubilized hepatocyte canalicular membrane. *Eur J Biochem* **224**: 345–352

Dantzig AH, Shepard RL, Cao J, Law KL, Ehlhardt, WJ, Baughman TM, Bumol TF and Starling JJ (1996) Reversal of P-glycoprotein-mediated multidrug resistance by a potent cyclopropyldibenzosuberane modulator, LY335979. *Cancer Res* **56**: 4171–4179

Denzlinger C, Rapp S, Hagmann W and Keppler D (1985) Leukotrienes as mediators in tissue trauma. *Science* **230**: 330–332

Devault A and Gros P (1990) Two members of the mouse mdr family confer drug resistance with overlapping but distinct drug specificities. *Mol Cell Biol* **10**: 1652–1663

Ford JM and Hait WN (1990) Pharmacology of drugs that alter multidrug resistance in cancer. *Pharmacol Rev* **42**: 155–199

Ford JM, Prozialeck W and Hait WN (1989) Structural features determining activity of phenothiazines and related drugs for inhibition of cell growth and reversal of multidrug resistance. *Mol Pharmacol* **35**: 105–115

Gerloff T, Stieger B, Hagenbuch B, Madon J, Landmann L, Roth J, Hofmann AF and Meier PJ (1998) The sister of P-glycoprotein represents the canalicular bile salt export pump of mammalian liver. *J Biol Chem* **273**: 10046–10050

Gottesman MM and Pastan I (1993) Biochemistry of multidrug resistance mediated by the multidrug transporter. *Annu Rev Biochem* **62**: 385–427

Gros P, Raymond M, Bell J and Housman D (1988) Cloning and characterization of a second member of the mouse *mdr* gene family. *Mol Cell Biol* **8**: 2770–2778

Guhlmann A, Krauss K, Oberdorfer F, Siegel T, Scheuber PH, Müller J, Csuk-Glänzer B, Ziegler S, Ostertag H and Keppler D (1995) Noninvasive assessment of hepatobiliary and renal elimination of cysteinyl leukotrienes by positron emission tomography. *Hepatology* **21**: 1568–1575

Hait WN and Aftab DT (1992) Rational design and pre-clinical pharmacology of drugs for reversing multidrug resistance. *Biochem Pharmacol* **43**: 103–107

Hyafil F, Vergely C, Du Vignaud P and Grand-Perret T (1993) In vitro and in vivo reversal of multidrug resistance by GF120918, an acridone carboxamide derivative. *Cancer Res* **53**: 4595–4602

Ishikawa T, Müller M, Klünemann C, Schaub T and Keppler D (1990) ATP-dependent primary active transport of cysteinyl leukotrienes across the liver canalicular membrane. Role of the ATP-dependent transport system for glutathione *S*-conjugates. *J Biol Chem* **265**: 19279–19286

Juliano RL and Ling V (1976) A surface glycoprotein modulating drug permeability in Chinese hamster ovary cell mutants. *Biochim Biophys Acta* **455**: 152–162

Kadmon M, Klünemann C, Böhme M, Ishikawa T, Gorgas K, Otto G, Herfarth C and Keppler D (1993) Inhibition by cyclosporin A of adenosine triphosphate-dependent transport from the hepatocyte into bile. *Gastroenterology* **104**: 1507–1514

Kamimoto Y, Gatmaitan Z, Hsu J and Arias IM (1989) The function of the Gp170, the multidrug resistance gene product, in rat liver canalicular membranes. *J Biol Chem* **264**: 11693–11698

Keppler D and König J (1997) Expression and localization of the conjugate export pump encoded by the *MRP2* (*cMRP/cMOAT*) gene in liver. *FASEB J* **11**: 509–616

Keppler D, Jedlitschky G and Leier I (1998) Transport function and substrate specificity of multidrug resistance protein. *Methods Enzymol* **292**: 607–616

Laemmli UK (1970) Cleavage of structural proteins during the assembly of the head of bacteriophage T4. *Nature* **227**: 680–685

Leier I, Jedlitschky G, Buchholz U and Keppler D (1994) Characterization of the ATP-dependent leukotriene $C_4$ export carrier in mastocytoma cells. *Eur J Biochem* **220**: 599–606

Lin MC, Kramer W and Wilson FA (1990) Identification of cytosolic and microsomal bile acid-binding proteins in rat ileal enterocytes. *J Biol Chem* **265**: 14986–14990

Loe DW, Deeley RG and Cole SPC (1996) Biology of the multidrug resistance-associated protein, MRP. *Eur J Cancer* **32A**: 945–957

Meier PJ and Boyer JL (1990) Preparation of basolateral (sinusoidal) and canalicular plasma membrane vesicles for the study of hepatic transport processes. *Methods Enzymol* **192**: 534–545

Müller M, Ishikawa T, Berger U, Klünemann C, Lucka L, Schreyer A, Kannicht C, Reutter W, Kurz G and Keppler D (1991) ATP-dependent transport of taurocholate across the hepatocyte canalicular membrane mediated by a 110-kDa glycoprotein binding ATP and bile salt. *J Biol Chem* **266**: 18920–18926

© Cancer Research Campaign 1999 

Müller M, Mayer R, Hero U and Keppler D (1994*a*) ATP-dependent transport of amphiphilic cations across the hepatocyte canalicular membrane mediated by mdr 1 P-glycoprotein. *FEBS Lett* **343**: 168–172

Müller M, Mittenbühler K, Mayer R, Wallstab A, Silverman JA, Thorgeiersson SS and Keppler D (1994*b*) ATP-dependent transport of amphiphilic cations across the canalicular membrane mediated by P-glycoprotein. In *Transport in the Liver.* Keppler D and Jungermann K (eds), pp 156–165, Academic Publishers: Dordrecht

Nicholson DW, Klemba MW, Rasper DM, Metters KM, Zamboni RJ and Ford-Hutchinson RW (1992) Purification of human leukotriene C4 synthase from dimethylsulfoxide-differentiated U937 cells. *Eur J Biochem* **209**: 725–734

Nishida T, Gatmaitan Z, Che MX and Arias IM (1991) Rat liver canalicular membrane vesicles containing an ATP-dependent bile acid transport system. *Proc Natl Acad Sci USA* **88**: 6590–6594

Pearce HL, Safa AR, Bach NJ, Winzer MA, Cirtain MC and Beck WT (1989) Essential features of the P-glycoprotein pharmacophore as defined by a series of reserpine analogs modulate multidrug resistance. *Proc Natl Acad Sci USA* **86**: 5128–5132

Pennock GD, Dalton WS, Roeske WR, Appleton CP, Mosley K, Plezia P, Miller TP and Salmon SE (1991) Systemic toxic effects associated with high-dose verapamil infusion and chemotherapy administration. *J Natl Cancer Inst* **83**: 105–110

Roninson IB, Chin JE, Choi K, Gros P, Housman DE, Fojo A, Shen D-W, Gottesman MM and Pastan I (1986) Isolation of human mdr DNA sequences amplified in multidrug-resistant KB carcinoma cells. *Proc Natl Acad Sci USA* **83**: 4538–4542

Schinkel AH, Smit JJM, van Tellingen O, Beijnen JH, Wagenaar E, van Deemter L, Mol CAAM, van der Valk MA, Robanus-Maandag EC, le Riele HP, Berns AJM and Borst P (1994) Disruption of the mouse *mdr1a* P-glycoprotein gene leads to a deficiency in the blood–brain barrier and to increased sensitivity to drugs. *Cell* **77**: 491–502

Thiebaut F, Tsuruo T, Hamada H, Gottesman MM, Pastan I and Willingham MC (1987) Cellular localization of the multidrug-resistance gene product P-glycoprotein in normal human tissues. *Proc Natl Acad Sci USA* **84**: 7735–7738

Tsuruo T, Iida H, Tsukagoshi S and Sakurai Y (1981) Overcoming of vincristine resistance in P833 leukemia in vivo and in vitro through enhanced cytotoxicity of vincristine and vinblastine by verapamil. *Cancer Res* **41**: 1967–1972

Twentyman PR (1992) Cyclosporins as multidrug resistance modifiers. *Biochem Pharmacol* **43**: 109–117

Watanabe T, Tsuge H, Oh-Hara T, Naito M and Tsuruo T (1995) Comparative study on reversal efficiency of SDZ PSC 833, cyclosporin A and verapamil on multidrug resistance in vitro and in vivo. *Acta Oncol* **34**: 235–241

Yang CP-H, Cohen D, Greenberger LM, Hsu SI-H and Horwitz SB (1990) Differential transport properties of two *mdr* gene products are distinguished by progesterone. *J Biol Chem* **265**: 10282–1028

*© Cancer Research Campaign 1999*
